# EGFR inhibitor-induced folliculitis decalvans: a case series and management guidelines

**DOI:** 10.1097/CAD.0000000000001494

**Published:** 2023-01-24

**Authors:** Joanna Nowaczyk, Kamil Fret, Grazyna Kaminska-Winciorek, Lidia Rudnicka, Joanna Czuwara

**Affiliations:** a Department of Dermatology, Medical University of Warsaw, Warsaw; b Department of Bone Marrow Transplantation and Onco-Haematology, Maria Sklodowska-Curie National Research Institute of Oncology (MSCNRIO), Gliwice Branch, Poland

**Keywords:** adverse events, afatinib, case series, epidermal growth factor receptor-inhibitor, folliculitis decalvans, management guidelines, skin toxicity

## Abstract

Epidermal growth factor receptor (EGFR) is one of therapeutic targets in oncology for solid tumors originating from epithelial tissue, such as non-small-cell lung carcinoma (NSCLC) and breast cancer. EGFR inhibitors used in cancer treatment may cause a broad spectrum of dose-dependent cutaneous adverse events, including acneiform papulopustular rash, nail and hair disturbances, xerosis, and mucositis. The pathogenesis of the EGFR inhibitor-induced adverse reactions originates from disturbances in keratinocyte differentiation, cytokine secretion, and neutrophil chemotaxis. One of the rare, yet distressing adverse events may be folliculitis decalvans, a progressive neutrophil-driven scarring alopecia with hair tufts formation resembling doll’s hair. Early diagnosis and introduction of treatment are crucial for disease prognosis since a long course of the disease leads to decreased quality of life. Here, we review the literature cases of EGFR inhibitor-induced folliculitis decalvans and provide guidance on management and prevention of this condition in oncologic patients. Furthermore, we report the first afatinib-associated folliculitis decalvans in three female patients with NSCLC.

## Introduction

Epidermal growth factor receptor (EGFR) plays a crucial role in the pathways of cell division, apoptosis, and epithelial differentiation [1]. This receptor became a therapeutic target in oncology when a connection between its overexpression and processes of proliferation, angiogenesis, and metastasis in cancers has been found [1]. Three types of anticancer therapeutics inhibiting EGFR are used in clinical practice – monoclonal antibodies (such as cetuximab and panitumumab), tyrosine kinase inhibitors (such as gefitinib, erlotinib), or multispecific tyrosine kinase such as EGFR/HER2 inhibitors (afatinib, neratinib, and lapatinib). The latter acts against EGFR tyrosine kinase and also against vascular endothelial growth factor receptor (VEGFR) and rearranged during transfection (RET) signaling. Among solid tumors that may be qualified for this therapy are those originating from the epithelial tissue, for example, non-small cell lung carcinoma (NSCLC), head and neck squamous cell carcinoma (HNSCC), and breast cancer [1].

**Fig. 1:**
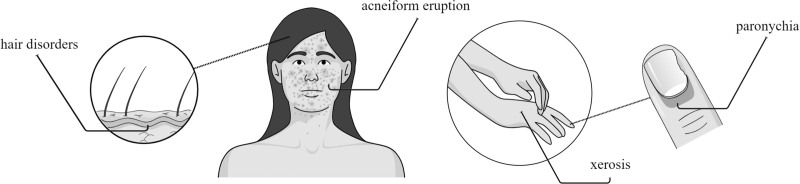
Common cutaneous adverse reactions of EGFR inhibitors. EGFR, epidermal growth factor receptor.

EGFR is found not only in epidermal cells but also in other dermal structures [1]. This may explain the presence of dose-dependent dermatological side effects of anti-EGFR therapy [1,2], which may affect the patient’s quality of life [3]. Most common adverse events are acneiform papulopustular rash (affecting 30–80% of patients), xerosis (20–50%), and paronychia (3–30%) [1] (Fig. 1). Hair conditions appear as late-side effects, affecting 1–5% of patients [1]. A very rare complication is folliculitis decalvans, a type of neutrophilic cicatricial alopecia most commonly occupying the vertex of the scalp [4]. If left untreated, this disease may cause progressive and irreversible hair loss due to hair follicle destruction.

## Case series

We included a consecutive series of three patients who were diagnosed and treated for folliculitis decalvans while on afatinib therapy from 2019 to 2020 in two dermatologic reference centers in Poland.

### Case I

**Fig. 2:**
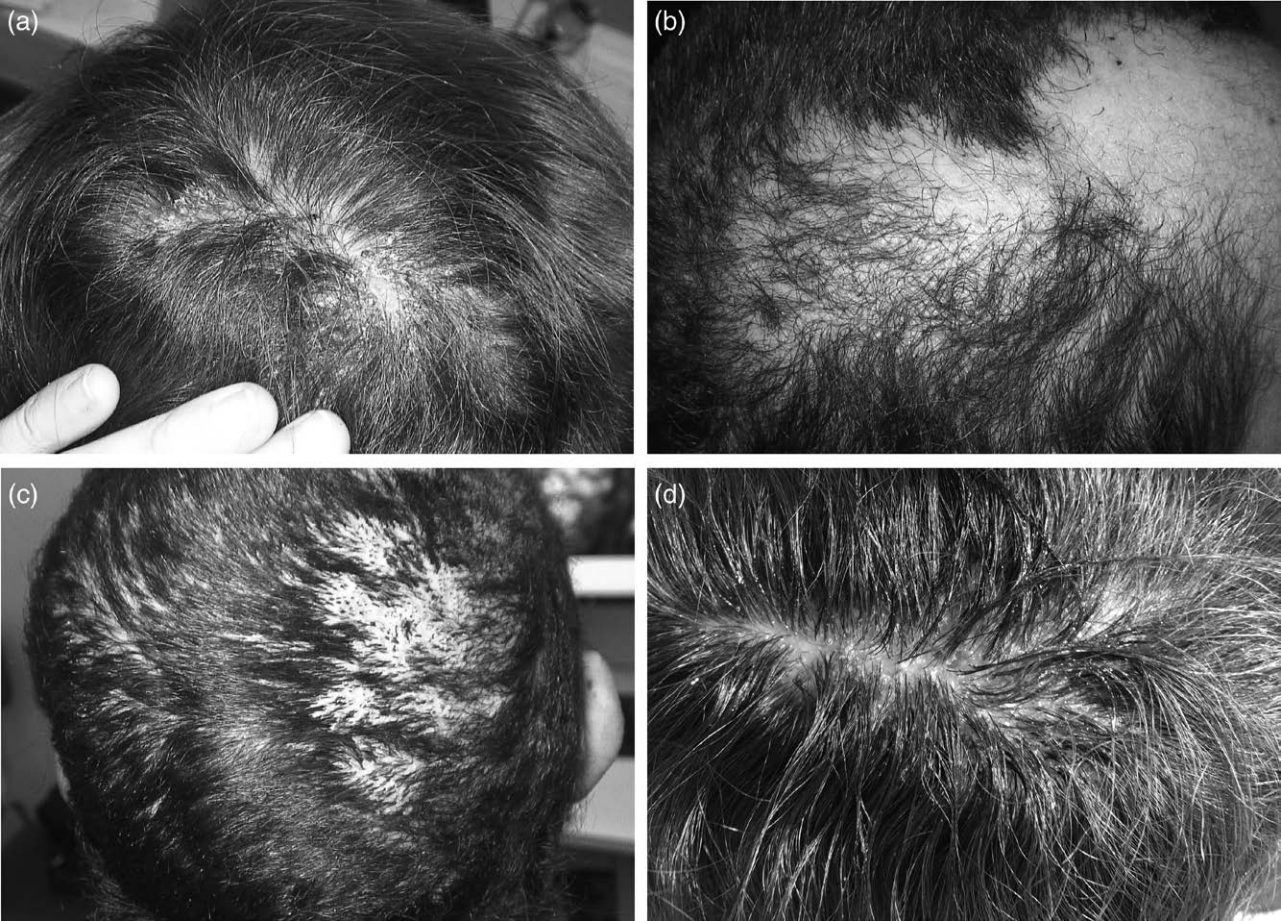
Macroscopic image of the first (a), second (b and c), and third patient (d). (a) Yellow crusts, extravasations, erythema, and alopecia on the vertex of the scalp (first patient). (b) Pili torti and atrichous areas on the frontal area (second patient). (c) Tufted hair and alopecia on the parieto-occipital area (second patient). (d) Yellow crusts, extravasations, erythema, and alopecia on the vertex of the scalp (third patient).

**Fig. 3:**
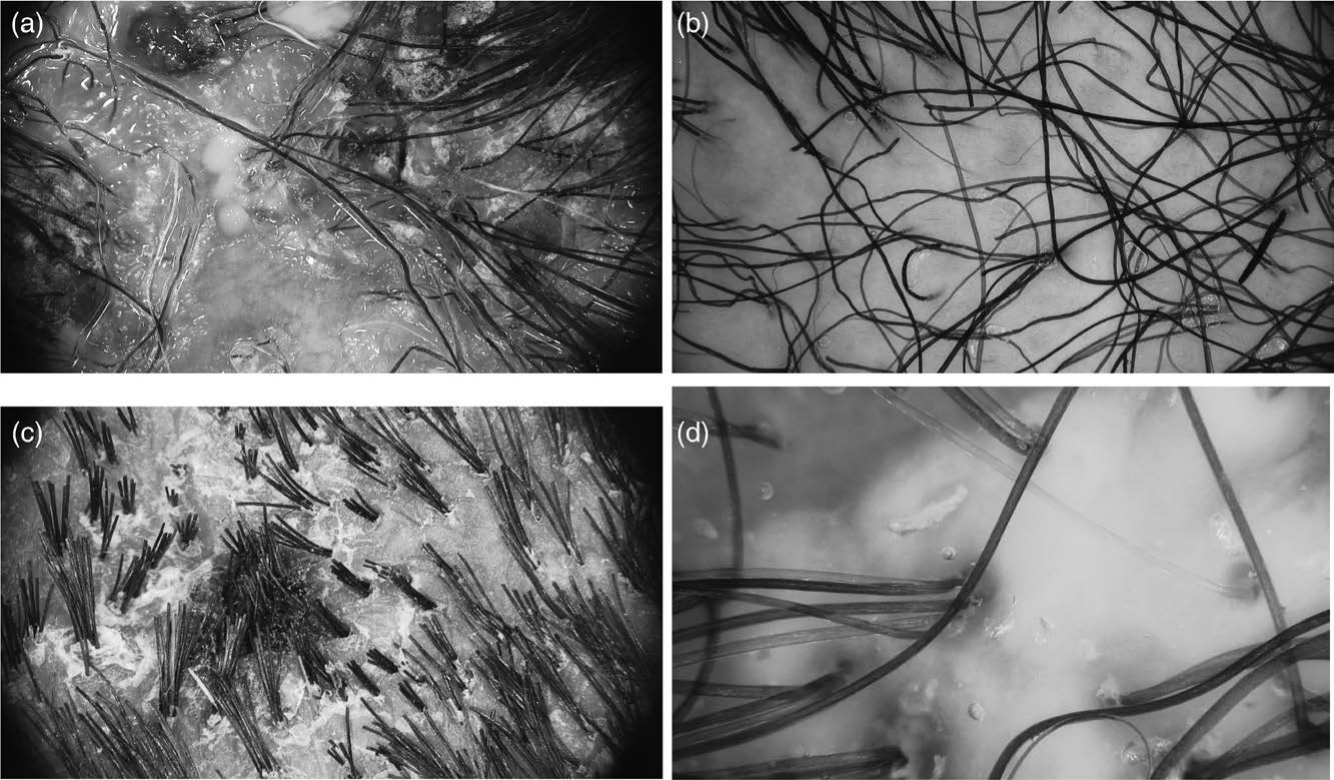
Trichoscopy of the first (a), second (b and c), and third patient (d). (a) Yellow crusts, whitish purulent discharge, extravasations, and pili torti (first patient). (b) Erythematous base and pili torti (second patient). (c) Hair tufts with polytrichia, tubular scaling formation, and crusts (second patient). (d) Hair tufts with polytrichia, erythematous base, and follicular pustules (third patient).

A 65-year-old Caucasian woman presented to the dermatological clinic due to scalp pruritus, rigid hair, and moderate hair loss. She had been taking afatinib for lung adenocarcinoma for 5 months. The woman also experienced facial erythema, nummular dermatitis, paronychia, and hyperkeratosis of her palms and soles. Treatment for hair loss was not recommended at that point, while adequate treatment was applied for the remaining conditions. Four months later, the woman returned with progressive alopecia with yellow crusting on the vertex, which started over two months earlier (Fig. 2a). Moreover, pyogenic granuloma of the index finger and inflammation of palms and soles were observed. Trichoscopy findings of the scalp included diffuse erythema, thin arborizing vessels, pili torti, yellow crusts, and purulent discharge (Fig. 3a). Based on these characteristic findings, folliculitis decalvans was diagnosed.

The patient received tetracycline (500 mg twice daily) and topical hydrocortisone butyrate. Improvement was observed after 2 months and the dosage of tetracycline was reduced to 250 mg twice daily.

### Case II

A 43-year-old Caucasian woman was referred to the dermatology clinic due to 1-month history of progressive folliculitis of her scalp. The patient had been undergoing treatment with afatinib (30 mg per day) for NSCLC. Physical examination of the scalp revealed hair loss, erythema, as well as tufted and twisted hairs on the frontal and parieto-occipital regions (Fig. 2b and c). Moreover, the patient experienced paronychia and acneiform papulopustular facial rash. Prior to the dermatology admission, the scalp lesions were treated with doxycycline and ciprofloxacin without improvement. Trichoscopic examination of the scalp revealed hair tufts, polytrichia, pili torti, crusts, scales, and blood extravasation (Fig. 3b and c). On this basis, folliculitis devalvans was diagnosed.

Initially, the scalp lesions were unsuccessfully treated with tetracycline (500 mg twice daily, then 750 mg twice daily) for one month. Improvement was finally achieved after 1-month of therapy with doxycycline (100 mg twice daily) combined with topical clobetasol propionate and hydrocortisone butyrate. After 2 months after the first appointment, neither scales nor crusts were present in trichoscopy; however, some purulent discharge was persistent. Therefore, maintenance therapy with doxycycline (100 mg once daily), topical hydrocortisone butyrate, and topical mupirocin was introduced.

### Case III

A 38-year-old Caucasian woman was consulted by a dermatologist due to 1-month history of severe pustular lesions located on her scalp, accompanied by pruritus and partial hair loss. For the last 24 months, the patient had been treated with afatinib for lung adenocarcinoma. Due to pneumonia, since the 15th week of the afatinib therapy, the woman had been treated at the altered dose of 20 mg once a day. In physical examination of the scalp, erythema and yellow crusts were observed on the vertex (Fig. 2d). In trichoscopy, the lesions had an appearance of perifollicular pustules with a tendency to form purulent and dried scabs (Fig. 3d). In microbiological examination of the scalp swab, methicillin-sensitive *Staphylococcus aureus* was cultured, with a sensitivity for doxycycline, fusidic acid, and amoxycillin with clavulanic acid. On the basis of these findings, folliculitis devalvans was diagnosed.

**Fig. 4:**
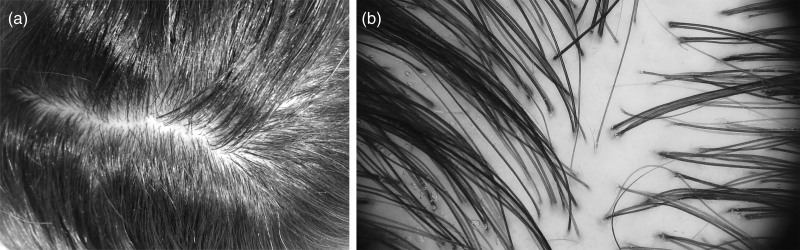
Clinical and trichoscopic improvement of the third patient. (a) Complete resolution of the scalp lesions (compare to Fig. 2d). (b) Trichoscopic findings characteristic for folliculitis decalvans are not present (compare to Fig. 3d).

Doxycycline was administered at a dose of 100 mg twice daily for 5 days, followed by 100 mg daily, and then tapered to 50 mg four times a day for the next 20 days owing to gastric symptoms. Additionally, mupirocin and betamethasone valerate with fusidic acid was prescribed topically. An improvement was noted 35 days later, with a total resolution of pustules and scaling (Fig. 4a and b).

## Discussion

To the best of our knowledge, this is the first case series of patients who developed folliculitis decalvans during afatinib therapy. Our three patients join the group of a few described cases of folliculitis decalvans during anti-EGFR therapy, as summarised in Table 1, [2,5–11]. In the literature reported cases of folliculitis decalvans, the median time from the start of EGFR inhibitors to the first symptoms of alopecia was 4 months (range: 1–23 months). The hair loss was often preceded by a rash, which was usually the first observed adverse reaction [5,7,9,10]. The hair loss was often followed by palmoplantar dermatitis [6], paronychia [10], and pyogenic granuloma [6] appearing weeks later.

**Table 1:** Literature review of other case reports describing folliculitis decalvans as an adverse event of epidermal growth factor receptor inhibitors

Study	Age, sex	Cancer type	EGFRI	Dosage of EGFRI	*S. aureus* scalp infection	Final treatment of folliculitis decalvans	Improvement of folliculitis decalvans (time)
Anzengruber *et al*.	51 M	HNSCC	Cetuximab	500 mg per day	Yes	Topical corticosteroids (class III), antiseptics	Yes (after 3 weeks)
Dervout *et al*.	54 F	Pulmonary adenocarcinoma	Erlotinib	no data	No data	Amoxicillin+clavulanic acid, topical corticosteroids, EGFR inhibitor discontinuation	Yes
Ena *et al*.	56 F	Breast cancer	Lapatinib	1250 mg per day	No	EGFR inhibitor dose reduction (to 1000 mg per day)	yes
Hoekzema *et al*.	60 F	Metastatic NSCLC	Erlotinib	150 mg per day	Yes (MSSA)	Minocycline (100 mg per day), desoximetasone 0.25% emulsion (once daily); maintenance: minocycline (50 mg per day for 2 months)	Yes (within 3 weeks)
Keith *et al*.	76 M	Metastatic pulmonary adenocarcinoma	Erlotinib	no data, mainten-ance dose of 75 mg per day	No data	Oxytetracycline (500 mg twice daily), betamethasone 0.1% with clioquinol 3% cream, cetrimide 10% with undecanoic acid 1% shampoo	Yes
Rosman *et al*.	47 F	Breast cancer	Trastuzumab	No data	No data	Topical clobetasol propionate 0.05% (twice a day)	Yes (‘quick’)
Sahuquillo-Torralba *et al*.	66 M	Metastatic pulmonary adenocarcinoma	Erlotinib	150 mg per day	Yes (MSSA)	EGFRI dose reduction (to 100 mg per day), doxycycline (100 mg twice a day)	No
Shih *et al*.	72 F	NSCLC	Gefitinib	250 mg per day	Yes	Fluocinonide cream, fusidic acid, doxycycline (1 month)	No (improvement when EGFRI stopped)
			Ficlatuzumab	20 mg/kg every 2 weeks			
	73 F	NSCLC	Gefitinib	250 mg per day	Yes	Fluocinonide, desoximetasone ointment, fusidic acid cream, doxycycline (200 mg per day for 4 months), cephalexin (2 g per day for 1 month)	No
			Ficlatuzumab	20 mg/kg every 2 weeks			

EGFRI, EGFR inhibitor; F, female; HNSCC, head and neck squamous cell carcinoma; M, male; NSCLC, non-small cell lung carcinoma; *S. aureus, Staphylococcus aureus.*

### Pathogenesis

Cutaneous toxicity of EGFR inhibitors originates from the interfered processes of proper maturation and differentiation of keratinocytes, mostly in the seborrheic areas of the skin [1,12,13]. Furthermore, the chemoattractant activity of EGFR inhibitors causes neutrophil chemotaxis and leads to sterile folliculitis [4,12]. A subsequent bacterial superinfection often co-occurs as EGFR inhibitors impair the antimicrobial defense of the skin [12,13]. In patients previously treated with radiation therapy, EGFR inhibitor-induced acne-like rash was found to spare the previously irradiated body regions due to radiation-induced follicular loss [14–16].

### Diagnostic process

Quick diagnostic process is an important first step in management of folliculitis decalvans, as this condition may lead to progressive scarring and permanent hair follicle loss which meaningly affect the patient’s quality of life. Pain, itching, and burning associated with the disease also play a significant role in patient’s wellbeing [4]. However, itching and hair casts can also appear as a result of stress accompanying anticancer therapy and may not be associated with folliculitis decalvans [6].

In favor of noninvasiveness, simplicity of implementation, and widespread availability, the main diagnostic tool of folliculitis decalvans is a dermatoscope to perform trichoscopy. Trichoscopy is a diagnostic examination of the scalp surface, visualized at magnification and usually in polarised light. Characteristic signs of folliculitis decalvans in trichoscopy consist of hair tufts, yellow purulent discharge, perifollicular erythema, follicular hyperkeratosis, extravasations, pili torti, dystrophic hair, broken hair, and atrichous areas [4,17–20]. The term hair tufts concerns polytrichia, when 5–20 hairs become grouped into doll’s hair-like follicular bundles, as a result of grouping the destructed outer root sheaths of follicles by the neutrophil-rich inflammatory infiltrate to non-inflammatory areas [4,21,22].

If the trichoscopic examination fails to confirm a diagnosis, a punch biopsy taken from the pustular or papular lesions should be considered; however, this is not routinely required. Histopathological features indicating folliculitis decalvans are intrafollicular and perifollicular inflammatory infiltrates rich in neutrophils and plasma cells. Pustules form around the upper portion of the affected follicles. Epithelia of the follicles are hypertrophic with marked neutrophils exocytosis. With time, epithelia become destructed leading to naked hair shafts release and foreign body granulomas formation followed by fibrosis after resolution [2,5–9].

Bacterial superantigens of *S. aureus* may also be involved in the etiopathogenesis of folliculitis decalvans [4,23]. *S. aureus* is almost always obtained from microbiological culture of material collected from the lesions [4–7,9,10]. Treatment of superinfection is important to prevent the development of bacteriaemia and sepsis in immunocompromised patients [13].

### Management

Despite that typical and EGFR inhibitor-induced folliculitis decalvans have different backgrounds, for both types of the disease, a similar therapeutic approach with antimicrobial and immunomodulating medications can be applied.

Treatment of EGFR inhibitor-induced folliculitis decalvans frequently consists of oral tetracycline, topical steroids, and optional topical antibiotics (Table 2). Oral tetracyclines, especially doxycycline, are recommended in the treatment of folliculitis decalvans [4,24] due to their anti-inflammatory and antibacterial properties. Tetracyclines were also used in management of our patients and most of the literature-reported cases of folliculitis decalvans (Table 1). Therapeutic options for treatment induction include either doxycycline 100–200 mg twice daily, tetracycline 500–1000 mg twice daily, or minocycline 100 mg twice daily [25]. Therapy duration with tetracyclines should be continued for 4 up to 16 weeks; however, it may be prolonged to a few months or even years if the course of disease is recalcitrant or relapsing [25]. After marked improvement, medication can be tapered from the initially maximal, antibacterial doses to usually a half lower, anti-inflammatory doses for remission maintenance. Both EGFR inhibitors and tetracyclines require protection from UV radiation, therefore, patients must avoid sun exposure or use appropriate photoprotection (sun creams with sun protection factor, and clothing covering the skin) [26]. Treatment with tetracyclines is usually well-tolerated, although high doses may contribute to gastrointestinal upset or diarrhea, especially in regard to minocycline [27].

**Table 2:** Proposed management of anti-epidermal growth factor receptor inhibitor-induced folliculitis decalvans

Route	Group	First-line	Second-line
Oral		Doxycyline 100–200 mg twice daily	Rifampicin 150–300 mg twice daily with clindamycin 300 mg twice daily or daily.
	Antibiotics p.o.	Tetracycline 500–1000 mg twice daily	Isotretinoin 0.1–1.0 mg/kg/d or 30 mg/day
		Minocycline 100 mg twice daily	Dapsone 100 mg/day
	*AND*		
Topical		Hydrocortisone butyrate	
	Corticosteroids top.	Clobetasol propionate	
		Betamethasone valerate	
	*OR*		
		Fusidic acid	
	Antibiotics top.	Mupirocin	
		Erythromycin	
Intra-lesional	*OR*		
	Corticosteroids s.c.		Triamcinolone

Other systemic medications that show efficacy involve rifampicin (150–300 mg twice daily) with clindamycin (300 mg twice daily or daily), isotretinoin (0.1–1.0 mg/kg/day or 30 mg/day), and dapsone (100 mg/day) [4,24].

Topical antibiotics (such as fusidic acid, mupirocin, erythromycin) [4] and topical steroids (such as hydrocortisone butyrate, clobetasol propionate, and betamethasone valerate) [2,5] or intralesional triamcinolone [24,25] can be used adjuvantly to increase the efficacy of the treatment, but only in combination with the systemic therapy [7,8]. Topical steroids were applied in all three of our cases and additionally, in two of our cases (including the one with *S. aureus* detected) topical antibiotics were prescribed. Topical medications were also commonly used in the management of other literature-reported cases of folliculitis decalvans (Table 1).

Furthermore, patients should be advised to use gentle hair products and avoid harsh anti-dandruff shampoos, hot-air blow drying, and aggressive hair styling [26].

### Prevention

Recently, a proactive (preventive) approach to cutaneous toxicities of EGFR inhibitors has been studied to improve the patients’ quality of life, maintain treatment compliance, and avoid dose changes or interruptions of anti-EGFR therapy [28–30]. Pre-emptive use of low-dose systemic or topical tetracyclines [28,31,32] with topical steroids [29,33] for EGFR inhibitor-induced cutaneous adverse events have been analyzed in randomized open-label trials and a retrospective cohort study showing promising outcomes in reducing the incidence or severity of a rash but not necessarily other side effects, including hair disorders.

### Decision upon epidermal growth factor receptor-inhibitor dose reduction

One of the challenges in management of folliculitis decalvans is the resistance to the therapy [4] or disease recurrence. Ineffective treatment of severe or progressive folliculitis decalvans may require a dose reduction [6,10] or in critical cases even a discontinuation of the anti-cancer drug until improvement is required [11]. This decision should be made cautiously and primarily the control of the underlying oncological disease should be considered [9]. Some patients may also decide to abruptly stop the anti-EGFR therapy owing to severe cutaneous adverse events causing pain, itching, and cosmetic concerns. Therefore, it is important for the oncologists to inform the patient beforehand about these manageable side effects and refer to a dermatologist at early stage instead of in the case of persistence or worsening of the skin lesions [34].

## Acknowledgements

J.C. and G.K.W. provided clinical data. J.N. and K.F. analyzed the data and drafted the article. All authors participated in the discussion and revised the article.

Patients have given consent for publication.

### Conflicts of interest

There are no conflicts of interest.
